# Production and characterization of exopolysaccharides from *Pseudomonas aeruginosa* AG01 with some medical potential applications

**DOI:** 10.1186/s12934-025-02730-z

**Published:** 2025-05-14

**Authors:** Amira Mohamed Ghanaim, Heba I. Mohamed, Abeer E. El‑Ansary

**Affiliations:** 1 Biological and Geological Sciences Department, Faculty of Education, Ain Shams University, Cairo, 11341 Egypt; 2 Biochemistry Department, Faculty of Agriculture, Cairo University, Gamma St, Giza, 12613 Egypt

**Keywords:** FT-IR, HPLC, Antitumor, Antiviral, Antimicrobial, Antibiofilm, Antioxidant

## Abstract

There is growing interest in the production and characterization of bacterial exopolysaccharides (EPS) because of their diverse range of applications. This study aimed to examine the production of EPS by *Pseudomonas aeruginosa* AG01, to characterize the produced EPS and its application as antioxidant, antimicrobial, antibiofilm, antitumor, and antiviral activity. The results indicated that the ideal conditions for achieving the highest production of EPS included an incubation period of 96 h, a pH level of 6, and a temperature of 32 °C in a nutrient broth medium. The most efficient sources of carbon and nitrogen for the formation of EPS were found to be galactose, glucose, yeast extract, and peptone. Several functional groups were confirmed to be present by Fourier transform infrared spectroscopy including amino groups, amides, carboxylic acids, hydroxyl groups, and phosphates. In the same respect, EPS has antioxidant activity. Moreover, EPS produced by *Pseudomonas aeruginosa* AG01 demonstrated antibacterial activity against various Gram-positive, Gram-negative bacteria, and yeast, besides antibiofilm activity about 98.93%, 98.86%, 98.63%, and 97.19% for *Bacillus subtilis*,* Staphylococcus aureus*,* Klebsiella pneumoniae*, and *Escherichia coli*, respectively as well as anticancer activity against prostate cancer (PC3) and breast cancer (MCF7) cells with IC50 values of 156.41 and 156.41 µg/ml, respectively. Flow cytometry analysis revealed that MCF7 cells treated with EPS at a concentration of 500 µg/ml for 48 h showed a reduction in the percentage of cells in both the G0/G1 and S phases compared to the untreated control MCF7 cells. EPS resulted in apoptosis induction in MCF7 cells using the Annexin V-FITC PI staining method. The findings indicate that EPS demonstrates significant antiviral activity against both the herpes simplex virus (HSV-1) and the hepatitis A virus (HAV). In conclusion, EPS has great potential to be developed as a natural antioxidant or used in medicine and pharmaceuticals.

**Supplementary Information:**

The online version contains supplementary material available at 10.1186/s12934-025-02730-z.

## Introduction

The incidence of human diseases and infections is rapidly rising worldwide, including in industrialized countries. Antimicrobial resistance has emerged as one of the most significant public health challenges globally. This issue poses numerous public health problems, as many microbial infections are becoming resistant to traditional antimicrobial treatments [1]. Furthermore, viral infections are a major global issue that affects social life and the global economy directly or indirectly. They also cause significant morbidity. The need for new exploratory methods is highlighted by the rise in treatment resistance and the difficulties traditional medications have in curing hidden infections [2]. Regretfully, the successful vaccination approach is limited to specific virus species [3]. Additionally, creating good drugs is a drawn-out process that frequently fails, which is why there aren’t many antiviral drugs. When reactive oxygen species (ROS) are produced in excess, exceeding the body’s ability to eliminate or repair the damage, oxidative stress occurs [1]. Recent studies indicate that ROS and free radicals produced from oxygen can cause various harmful effects, including DNA damage, cancer development, and cellular degeneration. Additionally, these reactive species may contribute to the development of several chronic diseases, including coronary heart disease, carcinomas, and numerous other age-related cardiac issues [4–5]. These extremely reactive species, which include hydrogen peroxide, superoxide, and hydroxyl radicals, have been discovered as the primary cause of human health problems, such as cancer, atherosclerosis, rheumatoid arthritis, and neurological disorders [4]. These negative consequences result from reactive oxygen species’ actions and the degree of damage to biological molecules such as proteins, lipids, and DNA [4]. Researchers have been forced to look for natural antioxidants that don’t negatively impact human health because of growing concerns about the toxicity and safety of synthetic antioxidants [6–7]. Among these compounds, exopolysaccharides exhibit effective anticancer, antioxidant, antiviral, and immune-stimulating activities [8–12] due to their minimal side effects, such as cytotoxicity, which makes them suitable for use. This evidence has led to increasing interest in exopolysaccharides as potential sources for medicinal treatments.

Extracellular polysaccharides (EPSs) are a type of extracellular polymeric substance produced by various microorganisms, including bacteria, fungi, and microalgae [13]. Environmental factors and the type of microbes determine the composition of these complex macromolecules with a high molecular weight, known as polymers. They can be either homopolymeric or heteropolymeric in structure [14]. Homopolysaccharides can either be unbranched or branched and are made up of a single type of repeating unit consisting of monosaccharides, such as glucose and fructose, connected by glycosidic bonds. In contrast, heteropolysaccharides are usually branched and contain repeating units that consist of more than one type of monosaccharide [15].

EPSs can be secreted by various bacterial genera, facilitating cell attachment, adapting to the environment, and enhancing stress tolerance, all of which are crucial for the formation of microbial biofilms [16]. Many different types of bacteria like Gram-positive and Gram-negative bacteria create bacterial exopolysaccharides which have a variety of structures [17–18]. EPS chemical characteristics change according to growth conditions, nutrition levels, and culture conditions. Temperature and pH are two important variables that affect how EPS is formed and can enhance the enzymatic activity of EPS [19]. Furthermore, EPS chemical properties are mostly dictated by the availability and concentration of nutrients [20].

Recently, there has been a lot of interest in the application of bacterial exopolysaccharides in a variety of scientific, industrial, medical, and technological domains [16, 21]. Based on where they are found, bacterial exopolysaccharides are divided into two categories: capsular polysaccharides, which are firmly connected to the cell surface, and free slime polysaccharides, which are partially or fully released into the extracellular environment [15, 22]. Therefore, it is relatively easy and economical to extract them from the cell-free supernatant using these approaches [22]. EPS plays several essential roles for bacteria, including aiding cell adhesion, and cell aggregation, protecting against harsh environmental conditions, symbiosis with plants, preventing desiccation, nutrient compartmentation, and resisting antimicrobials [20]. In order to promote bacterial survival, adaptability, persistent colonization, and tolerance to biotic and abiotic stressors, EPS promotes the production of biofilms [16]. When present in the cell wall, extracellular polysaccharides (EPS) contribute to the structural integrity and protection of bacterial cells. The capsular EPS is the primary component that allows bacterial cells to adhere to surfaces, which often leads to the formation of biofilms [23].

*Pseudomonas aeruginosa* is a potentially fatal bacterium that can infect both plants and animals, including people with respiratory disorders, burn injuries, and cystic fibrosis [24]. *Pseudomonas* species are thought to produce many extracellular polymeric substances and they are found and isolated from different sites, such as industrial waste and activated sludge [25]. *P. aeruginosa* exopolysaccharides are widely used in various medical applications. They serve as antacids and stomach protectors for anti-reflux treatment, are employed in dental impressions, and are utilized as microspheres for drug delivery. Additionally, they are incorporated into fibers used in wound dressings and bandages to promote hemostasis. *Pseudomonas* strains have also been effectively applied in environmental remediation, particularly for the decontamination of waters, soils, and sediments polluted with heavy metals [26].

This study aims to produce exopolysaccharides (EPSs) from *Pseudomonas aeruginosa* AG01 LC586427 and to optimize the culture conditions for enhanced production under static conditions. Additionally, the research includes the characterization of EPS from *P. aeruginosa* AG01 LC586427 and an examination of their biological activities, including antioxidants, antimicrobial, antibiofilm, antitumor, and antiviral properties.

## Materials and methods

### Microorganisms and culture media

*P. aeruginosa* AG01 LC586427 was used in this study. Bacterial isolate was cultured on nutrient broth medium (100 ml) containing 2% glucose and incubated on a static incubator for 72 h at 35^o^C and pH 7.

### Extraction of EPS

EPS was extracted and purified according to the Lin et al. [27] method. First, the bacterial cells were extracted from the bacterial cultures by centrifuging them for 20 min at 5,000 rpm. Then, 4% (w/v) TCA was added and subsequently vortexed and centrifuged at 11,200 ×g, 4 °C for 30 min to precipitate proteins. Two volumes of 10% (w/v) trichloroacetic acid were added to the filtrate to precipitate the proteins. The mixture was then kept overnight at 4 °C under static conditions. Following this, it was centrifuged at 25,000 g for 20 min. Next, four volumes of pre-chilled 95% (v/v) ethanol were added to the supernatant. This mixture was further refrigerated at 4 °C for 24 h, after which it was centrifuged again at 25,000 g for 20 min at 4 °C to obtain the polysaccharide precipitates. The resulting pellet was lyophilized to produce dry EPS powder. To prepare the EPS powder for further use, it was dissolved in 5 ml of distilled water and dialyzed using a dialysis membrane. The solution was then concentrated and lyophilized to obtain dry powdered crude EPS, measured in grams per 100 milliliters.

The total carbohydrate content (%) of crude exopolysaccharides (EPS) was determined using the phenol-sulfuric acid method, with glucose (2 mg/ml) serving as the standard [28]. To prepare the sample, approximately 0.5 ml of the EPS solution was mixed with 0.5 ml of 6% (v/v) phenol in a test tube. Then, 2.5 ml of concentrated sulfuric acid was added to the mixture. After allowing the mixture to sit for 10 min, it was placed in a water bath at 30 °C for 20 min. The absorbance at 490 nm was measured using a spectrophotometer. By referencing a standard curve, the carbohydrate content was determined from the absorbance value. The protein content of EPS was determined using the Bradford method [29]. To measure the protein, 2.5 ml of the protein reagent, which consists of 50 ml containing 2.0 mg/ml Coomassie Brilliant Blue G-250 in 95% ethanol and 100 ml of 85% phosphoric acid, was added to 0.5 ml of the polysaccharide solution (2 mg/ml). After mixing, the absorbance was measured at 595 nm using a spectrophotometer. The protein content was calculated by substituting the absorbance values into a standard curve. To determine the lipid content, a 0.2 g sample of EPS was extracted using a chloroform-methanol mixture in a 2:1 ratio. The mixture was agitated vigorously, and the solvent phase was recovered through centrifugation at 12,745 × g (10,000 rpm) for 15 min. This extraction process was repeated three times. All the solvent phases were combined, evaporated, and dried under a vacuum. The lipid content was measured using gravimetric analysis, following the method outlined by Makkar and Cameotra [30].

The EPS was weighed to calculate the yield after the resultant pellet was dried for an entire night at 60 °C. The yields of biomass and exopolysaccharides were reported in grams per 100 milliliters after the EPS was extracted from 100 milliliters of bacterial culture [31].

### Optimization of the incubation period, pH, and temperature

To assess the effect of incubation time on the production of exopolysaccharides (EPS), an optimized inoculum size with an optical density of 1 was added to each flask containing 100 ml of nutrient broth medium. The flasks were incubated at 35 °C for different durations: 24, 48, 72, 96, and 121 h.

To examine the impact of varying pH levels on the formation of EPS, 100 milliliters of liquid nutrient broth were also altered to pH values between 3 and 10. Both 0.1 N HCl and 0.1 N NaOH were used to accomplish this. This was the ideal incubation interval, and the cultures of *P. aeruginosa* AG01 were cultured for 96 h at 35 °C. The influence of temperature on exopolysaccharide yield was examined by incubating a distinct culture for 96 h at different temperatures (20, 25, 30, 32, 35, 37, and 40 °C) at the ideal pH of 6. Each of the three experiments was carried out three times. The yield of biomass and exopolysaccharide was measured in grams per 100 ml for each experiment [32]. The stability study of purified EPS from *P. aeruginosa* AG01 was done, and the carbohydrate content was determined using the phenol-sulfuric acid method with glucose as a standard.

### Effect of different carbon sources and nitrogen sources on EPS production

To investigate the need for additional nutrients in EPS production, various carbon sources, specifically glucose, galactose, fructose, sucrose, maltose, mannose, raffinose, lactose, and starch, were individually added to the nutrient broth medium at a concentration of 1% (w/v). The mixtures were then incubated at 32 °C for 96 h.

Additionally, to assess the impact of different nitrogen sources on EPS production, various organic (yeast extract, peptone, and beef extract) and inorganic (sodium nitrate, ammonium nitrate, ammonium chloride, and ammonium sulfate) nitrogen sources were incorporated individually at a final concentration of 0.5%. This was done in the presence of 1% galactose, identified as the optimal carbon source, and the samples were incubated under the same conditions. Each of the two experiments was carried out three times, and the yields of biomass and exopolysaccharides were measured and reported in grams per 100 ml for each experiment [33].

### Characterization of EPS

#### HPLC analysis for EPS monosaccharide composition

**Fig. 1:**
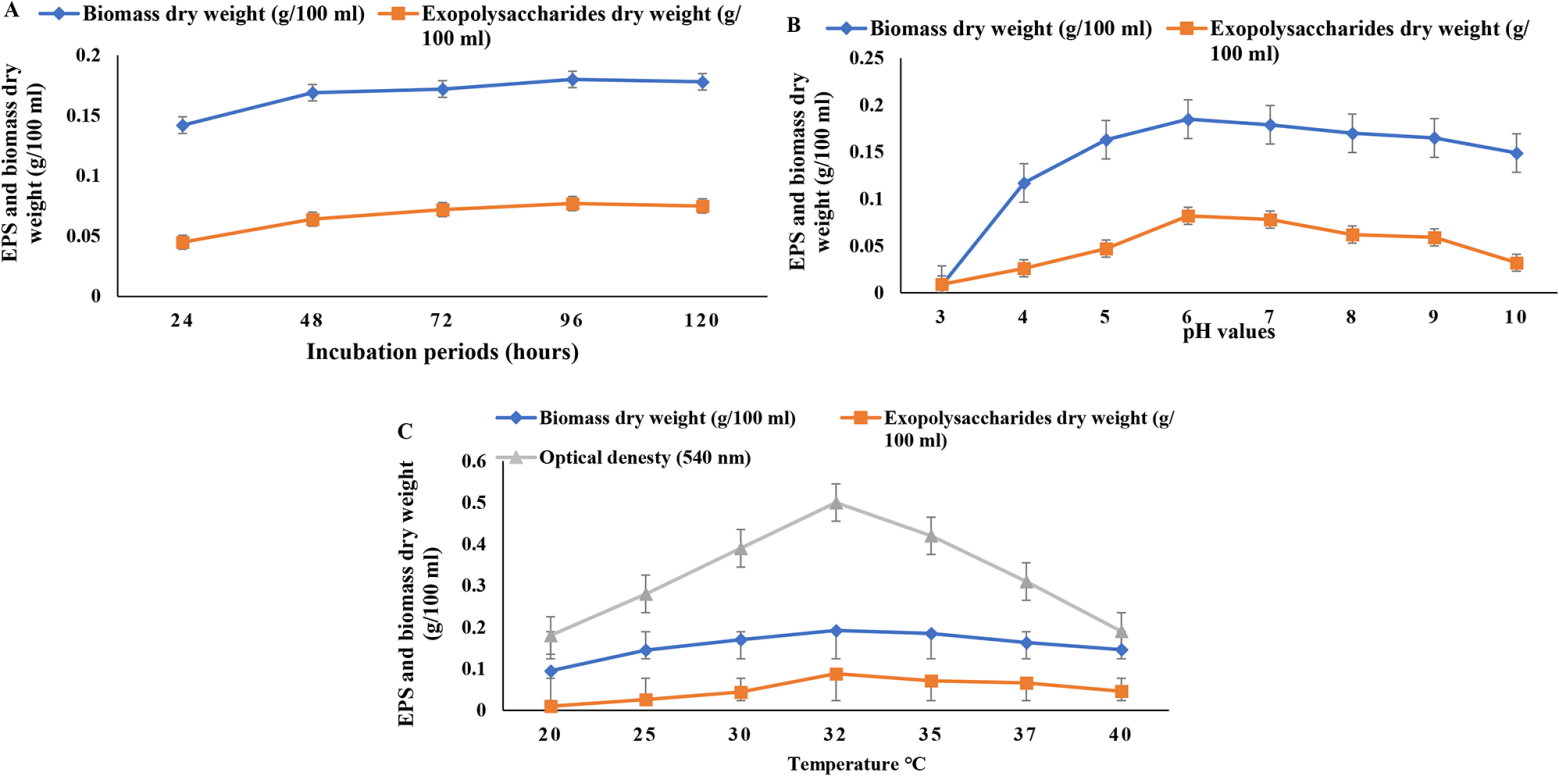
Effect of incubation periods (**A**), pH (**B**), and temperature (**C**) on bacterial biomass and EPS production by *Pseudomonas aeruginosa* in 100 ml culture media. The values are the means of three replicates with standard deviation (± SD)

Exopolysaccharides were hydrolyzed following the method described by Abou Zied et al. [34]. The carbohydrate content of the filtrate was analyzed using high-performance liquid chromatography (HPLC) with a Smart Line system from Knauer, Germany. Sugars were measured using a Phenomenex Luna NH_2_ column (250 × 4.6 mm) maintained at a temperature of 30 °C. The mobile phase consisted of 80% acetonitrile and 20% HPLC-grade water (v/v). Detection was performed using a refractive index (RI) detector, and data integration was conducted using ClarityChrom software. The environmental conditions during the experiment were a temperature of 20 °C and a relative humidity of 38%. The separated components were monitored using an ultraviolet (UV) detector at a wavelength of 254 nm. To determine the monosaccharide composition, the monosaccharide standards were used (Supplemented Fig. 1).

### Fourier transform infrared spectroscopy (FT-IR) analysis

To identify the functional groups in EPS, FT-IR was used. The investigation was performed using a Bruker Tensor 37 FT-IR spectrometer (FT-IR Nicolet 5700, Thermo Nicolet Co., Waltham, MA) that has a mercury cadmium telluride detector that was chilled by liquid nitrogen. A hydraulic press was used to compress a sample of 2 mg of dry EPS into pellets at a pressure of roughly 5–6 tons/cm² after it had been ground with about 200 mg of spectra-grade KBr (Sigma). The transmittance mode was used to measure the spectrum between 4000 and 400 cm⁻¹. The OPUS 3.1 program (Bruker Optics) was used to examine infrared (IR) spectra [35].

### Bio-applications of the produced EPS

#### Antioxidant activity (free radical-scavenging activity)

The 2,2-diphenyl-1-picrylhydrazyl (DPPH) method was used to evaluate the EPS antioxidant properties, as explained by Mohamed et al. [36]. The EPS sample from *P. aeruginosa* AG01 was tested for its capacity to scavenge free radicals by creating a reaction mixture. This mixture included 2 ml of a 0.2 mM DPPH solution in ethanol along with 2 ml of the EPS at different concentrations (100, 200, 300, and 400 µg/ml). After a thorough shake, the mixture was allowed to sit at room temperature for half an hour in the dark. The absorbance was measured at 517 nm using a spectrophotometer (Model 6305, Jenway, Staffordshire, United Kingdom) after this incubation. The mean values of the three experiments were computed. The antioxidant activity (radical scavenging activity) of the EPS was calculated according to the following equation:

$$\eqalign{& {\rm{\% DPPH = }} \cr & {\matrix{{\rm{Absrobance}}\,{\rm{at}}\,\,517\,{\rm{of}}\,{\rm{control}} \hfill \cr \,\,\,\,\,\,\,\,\,\,\,\,\,\,\, - {\rm{Absrobance}}\,{\rm{at}}\,\,517\,{\rm{of}}\,{\rm{sample}}\, \hfill \cr} \over {{\rm{Absrobance}}\,{\rm{at}}\,\,517\,{\rm{of}}\,{\rm{control}}}} \times 100 \cr} $$

An inhibitor’s 50% inhibition at the greatest concentration is known as the IC50 value.

### 2,2′‑Azino‑bis (3‑Ethylbenzothiazoline‑6‑Sulfonic Acid) (ABTS)

The decolorization of radical cations by ABTS was investigated according to Li et al. [37]. Five ml of 7 mM ABTS was mixed with 4.9 mM potassium persulfate to create the solution. The mixture was mixed with 1.8 ml of ABTS reagents and 0.2 ml of different quantities of purified EPS ethanolic extracts (varying from 100 to 400 µg/ml) after being left in the dark at room temperature for 16 h. The resulting mixture’s optical density was determined at 734 nm with a spectrophotometer (Model 6305, Jenway, Staffordshire, United Kingdom). 0.3 mM of L-ascorbic acid and butylated hydroxytoluene were utilized as a control. The following formula was used to determine the ABTS:

% Inhibition = ABS control − ABS Sample/ ABS control × 100.

### Antimicrobial activity

To evaluate the antimicrobial activity of theproduced EPS, Bauer et al. [38] used the agar well diffusion assay against a variety of microorganisms, including fungal strains (*Candida albicans* ATCC 10221 and *Aspergillus niger* ATCC 16888), Gram-positive bacteria (*Bacillus subtilis* ATCC 6633 and *Staphylococcus aureus* ATCC 6538), and Gram-negative bacteria (*Escherichia coli* ATCC 8739 and *Klebsiella pneumonia* ATCC13883) on Mueller-Hinton agar medium. A volume of the microbial inoculum was uniformly distributed over the whole agar surface to inoculate the agar plates. A sterile cork borer was then used to aseptically punch a 6 mm hole, and 100 µl of the EPS solution (50 mg) was added to the well. After that, the agar plates were incubated for 24 to 48 h at 30 to 37 °C for bacterial isolates and 24 to 72 h at 25 °C for fungal isolates. The diameter (in mm) of the inhibition zone encircling each EPS well was measured after incubation to determine the antimicrobial activity. Wells inoculated with Gentamicin (100 µg) and fluconazole (100 units) served as positive controls [39].

### Antibiofilm activity of EPS

#### Microtitre plate assay for biofilm quantification

The biofilm inhibitory activity of the extracted EPS was assessed using the microtitre plate assay (MTP) in 96-well polystyrene flat-bottom plates against four clinical microbes: *E. coli*,* P. aeruginosa*,* S. aureus*,* and B. subtilis*. The procedures followed were based on the methods outlined by Niu et al. [40] and Antunes et al. [41].

In brief, 100 µl of microbial suspensions at a concentration of 5 × 10^6 CFU/ml were mixed with the wells of the microplate, which contained 200 µl of TSYB. Biofilm formation was promoted by adding 0.5% glucose to the TSYB. After incubation at 35 °C for 24 h, serial concentrations of the tested EPS (1000, 500, 250, 125, 62.8, 1.95, 0.9, and 0.45 µg/ml) were added to the wells, followed by a 48-hour incubation at 37 °C.

Three wells containing bacterial suspension without EPS served as the growth control, while three wells containing media without bacterial inoculum were used as the blank control. After incubation, the supernatant was removed, and each well was washed thoroughly with sterile distilled water to eliminate free-floating cells. The plates were then air-dried for 30 min, and the biofilm that formed was stained for 15 min at room temperature with a 0.1% aqueous solution of crystal violet.

After staining, the excess dye was removed by washing the plates three times with sterile distilled water. Finally, the dye bound to the cells was solubilized by adding 250 µl of 95% ethanol to each well. After an additional 15 min of incubation, absorbance was measured using a microplate reader at a wavelength of 570 nm. Isolates that exhibited an optical density (O.D.) of 570 nm greater than 0.1 were considered positive for biofilm production.

$$\eqalign{& {\rm{Biofilm}}\,{\rm{inhibition}}\,{\rm{ability}}\,{\rm{of}}\,{\rm{sample}}\, \cr & \,\,\,\,\,\,\,1 - {{{\rm{Absorb}}.\,{\rm{sample}}\, - \,{\rm{Absorb}}.\,{\rm{Blank}}} \over {{\rm{Absorb}}.\,{\rm{sample}}\, - \,{\rm{Absorb}}.\,{\rm{Blank}}}} \times 100 \cr} $$

#### Cytotoxicity and antitumor activity in vitro

Following the methodology described by Slater et al. [42], the MTT (3-[4,5-dimethylthiazol-2-yl]-2,5-diphenyl tetrazolium bromide) assay was used to assess the cytotoxicity of EPS against normal Vero cell lines as well as their anticancer potential toward prostate cancer (PC-3) and breast cancer (MCF-7) cell lines. First, a full monolayer of cells was obtained by inoculating a 96-well tissue culture plate with 1 × 10^5^ cells/ml (100 µl per well) and incubating it for 24 h at 37 °C. Following the formation of the monolayer, the growth medium was taken out of the microtiter plates and the cell monolayer was washed twice with washing media. Tested samples were produced in RPMI medium with 2% serum (maintenance medium) in two-fold dilutions of different concentrations (1000, 500, 250, 125, 62.5, and 31.2 µg/ml). For each well 0.1 ml was added, whereas three wells served as controls and only one well received the maintenance medium. After that, the plate was incubated at 37 °C before being analyzed. Physical markers of toxicity within the cells were evaluated, searching for signals like granulation, shrinkage, rounding of the cells, or partial or whole loss of the monolayer. In each well, twenty microliters (µl) of BIO BASIC CANADA INC.‘s MTT solution (5 mg/ml in PBS) were applied.

The wells were then incubated for four hours at 37 °C in a humidified incubator with 5% CO_2_ after being shaken for five minutes at 150 rpm. The formazan, which is MTT’s metabolic product, was resuspended in 200 µl of DMSO after the medium was disposed of during incubation. Five more minutes were spent shaking the mixture at 150 rpm. Using an ELISA plate reader (BioTek Instrument, ELx808, USA), the optical density (OD) of the contents in the wells was measured at 560 nm, and background absorbance was subtracted at 620 nm. Using the following formulas, the percentage of inhibition and cell viability was determined.

Viability % = $$\:\frac{\mathbf{O}\mathbf{D}\:\mathbf{o}\mathbf{f}\:\mathbf{s}\mathbf{a}\mathbf{m}\mathbf{p}\mathbf{l}\mathbf{e}}{\mathbf{O}\mathbf{D}\:\mathbf{o}\mathbf{f}\:\mathbf{c}\mathbf{o}\mathbf{n}\mathbf{t}\mathbf{r}\mathbf{o}\mathbf{l}\:}\:$$× 100

Inhibition % = 100 − Viability %.

The IC50, or 50% inhibitory concentration, was calculated using graphical plots. As controls, undamaged cells were used in the tests, and the assays were carried out three times.

### Cell cycle analysis by flow cytometry

The effect of EPS on the cell cycle of breast cancer MCF7 cells was analyzed using flow cytometry. A flow cytometer measured the behavior of the cells in the presence of the extracted EPS. In brief, concerned cancer cells were seeded into 3.5 cm Petri dishes at a density of 2 × 10^5^ cells/dish. After collecting and washing MCF7 cells with cold phosphate-buffered saline (PBS), both EPS-treated and untreated cells were examined. Fluorescence-activated cell sorting (FACS) analysis was used to determine the amount of DNA in an aliquot of the cell suspension (10^5 cells/100 µl) by mixing it with 1 µl of fluorescein isothiocyanate (FITC)-conjugated annexin V and 2.5 µl of propidium iodide (PI) at a concentration of 500 µg/ml. We calculated the proportions of cells in the G1, S, and G2 phases of the cell cycle [43].

### Annexin-V FITC apoptotic assay

Using Annexin V-FITC, a flow cytometric examination of early and late apoptosis was carried out by Eldehna et al. [44]. Breast cancer (MCF7) cell lines were treated with EPS after being cultivated until they formed a confluent monolayer. Following a full day of treatment, the cells were taken out and given two 20-minute washes with phosphate-buffered saline (PBS) before being treated with the binding buffer. The cells were then treated with 1 µl of Annexin V-FITC for 40 min at 4 °C after being resuspended in 100 µl of the kit’s binding buffer. Following incubation, 1 µl of 4’,6-diamidino-2-phenylindole (DAPI) at a concentration of 1 µg/ml in PBS was added to 150 µl of binding buffer while the cells were once again washed and resuspended. Finally, a BD FACS Calibur flow cytometer was used to examine the cells.

### Antiviral assay

The antiviral activities of polysaccharides extracted from *P. aeruginosa* AG01 LC586427 were examined against the HSV-1 and HAV-2 viruses, obtained from the Faculty of Medicine for Girls, Microbiology Department (Cairo, Egypt). In this investigation, 10,000 cells were plated in a 96-well plate with 200 µl of medium per well. Three wells were used as blank controls and were left empty. The cells were allowed to attach to the wells by incubating the plate for a whole night at 37 °C with 5% CO_2_. The viral solution and the EPS sample under examination were incubated for one hour in a non-lethal dilution of the same volume (1:1 v/v). Add 100 µl of the viral/ EPS sample suspension so that the virus can begin to function. It should be incubated for 24 h at 37 °C with 5% CO_2_ after being placed on a shaking table for 5 min at 150 rpm. Make sure that each 96-well plate has at least two milliliters of MTT solution, with a concentration of 5 mg/ml in PBS. Each well should then contain 20 µl of MTT solution. The MTT was spun at 150 rpm for five minutes on a shaking table to completely integrate it into the media. To facilitate metabolism, the MTT reagent was incubated at 37˚C with 5% CO_2_ for one to five hours. If any residue needs to be eliminated, drain the medium (dry plate) using tissue paper. Formazan, the MTT metabolic product, was reconstituted in 200 µl of DMSO and shaken for five minutes at 150 rpm to mix the two. The optical density was measured at around 560 nm, and interference was removed at around 620 nm. There should be a correlation between optical density and cell number [45–46].

### Statistical analysis

Each value was expressed as the average of three replicates. Using the SPSS statistical software (SAS Institute Inc., Cary, NC), the data were examined using Duncan’s multiple range tests after analysis of variance (ANOVA).

## Results

### Effect of different growth conditions on biomass and EPS production

To determine the optimal conditions for maximum EPS production from *P. aeruginos*a AG01, we studied the effects of the incubation period, pH, temperature, and carbon and nitrogen sources.

### Effect of the different incubation periods

The production of EPS is affected by the duration of the incubation periods. The results in Fig. 1A showed that the bacterial biomass and EPS yield were increased by increasing the incubation periods up to 96 h and decreased slightly at 120 h. The highest yield of EPS was about 0.077 g/100 ml and the biomass of about 0.180 g/100 ml was recorded after 96 h.

### Effect of different pH values

The optimal pH for EPS production was 6, yielding approximately 0.0828 g/100 ml of EPS. Additionally, the dry weight of bacterial biomass from *P. aeruginosa* AG01 was around 0.185 g/100 ml after incubation at 35ºC for 96 h, identified as the optimal incubation period, as shown in Fig. 1B. It was observed that both EPS yield and bacterial biomass increased from pH 3 to 6 and then gradually declined as pH values increased above 6.

### Effect of different temperatures

After 96 h, *P. aeruginosa* AG01 cultures were incubated at various temperatures between 20 and 40 °C to determine the ideal temperature for the maximum EPS yield. The results in Fig. 1C illustrated that 32 ℃ is the optimum temperature to obtain the highest EPS yield (0.088 g/100 ml), bacterial biomass (0.192 g/100 ml), and the carbohydrate content (0.5 g/100 ml). We observed that growth and EPS yield decrease when temperatures exceed 32 °C. We measured the optical density at 540 nm. Our findings indicate that any further increase or decrease in temperature above 32 °C results in a decline in both growth and EPS yield. The stability of the exopolysaccharide was studied at the optimum temperature (32 °C) for 10 days *P. aeruginosa* AG01 exopolysaccharide was found to be quite stable.

### Effect of different carbon and nitrogen sources

**Fig. 2:**
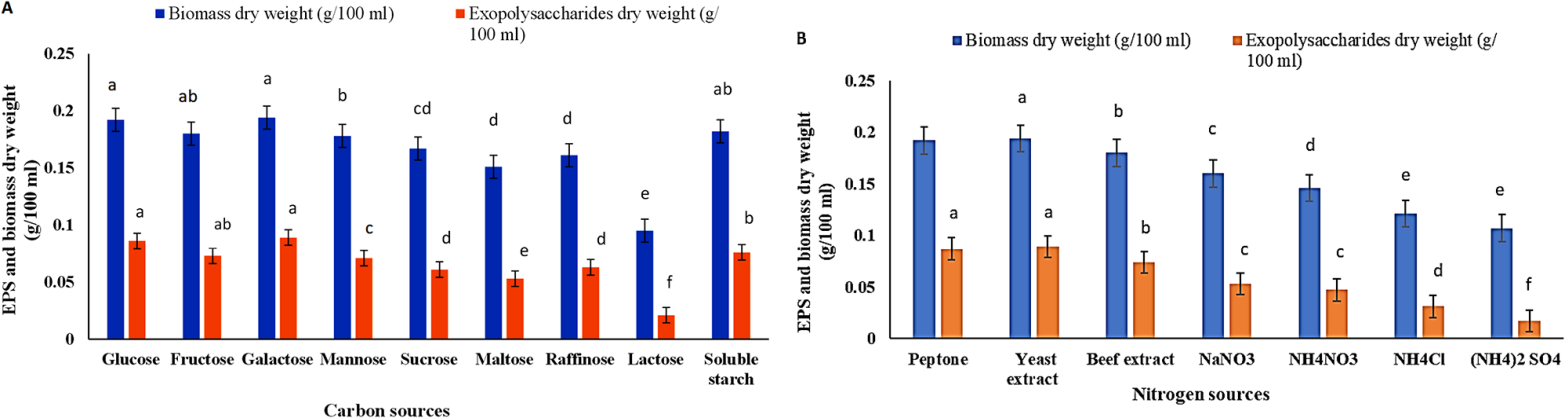
Effect of different carbon (**A**) and nitrogen (**B**) sources on bacterial biomass and EPS production by *Pseudomonas aeruginosa* in 100 mL culture media. The values are the means of three replicates with standard deviation (± SD). Mean values in each column followed by a different lower-case letter are significantly different according to Duncan’s multiple range tests at *p* ≤ 0.05

The data in Fig. 2A demonstrated that galactose and glucose were the most appropriate carbon sources, resulting in the maximum production of EPS produced by *P. aeruginosa* AG01. The EPS yield in galactose and glucose was 0.089 g/ml and 0.086 g/ml, and the biomass dry weight was 0.194 g/100 ml and 0.192 g/100 ml, respectively. However, media containing lactose showed the lowest production of EPS and biomass, by approximately 0.021 g/100 ml and 0.095 g/100 ml, respectively. The results of the effect of different nitrogen sources on the yield of EPS showed that the optimum production of EPS obtained by using yeast extract and peptone as a nitrogen source and recorded about 0.089 g/100 ml, and 0.087 g/100 ml, respectively and bacterial biomass was about 0.194 g/100 ml, and 0.192 g/100 ml, respectively followed by beef extract, while the lowest production of EPS and biomass recorded in media containing ammonium sulphate that recorded about 0.107 g/ 100 ml and 0.017 g/ 100 ml, respectively as illustrated in Fig. 2B.

### Chemical composition and characterization of EPS

The obtained EPS had a chemical composition of 511.6 mg/g carbohydrate, 28.0 mg/g protein, and 2.2 mg/g lipid (Table 1).

**Table 1:** Chemical composition of EPS produced by *P. aeruginosa* AG01

Chemical composition (mg/g)	Values
Total carbohydrates	511.6 ± 0.9
Total protein	28.0 ± 0.6
Total lipids	2.2 ± 0.06

The values are the means of three replicates with standard deviation (± SD)

### Characterization of EPS

#### Fourier transform Infra-red (FT-IR) analysis

**Fig. 3:**
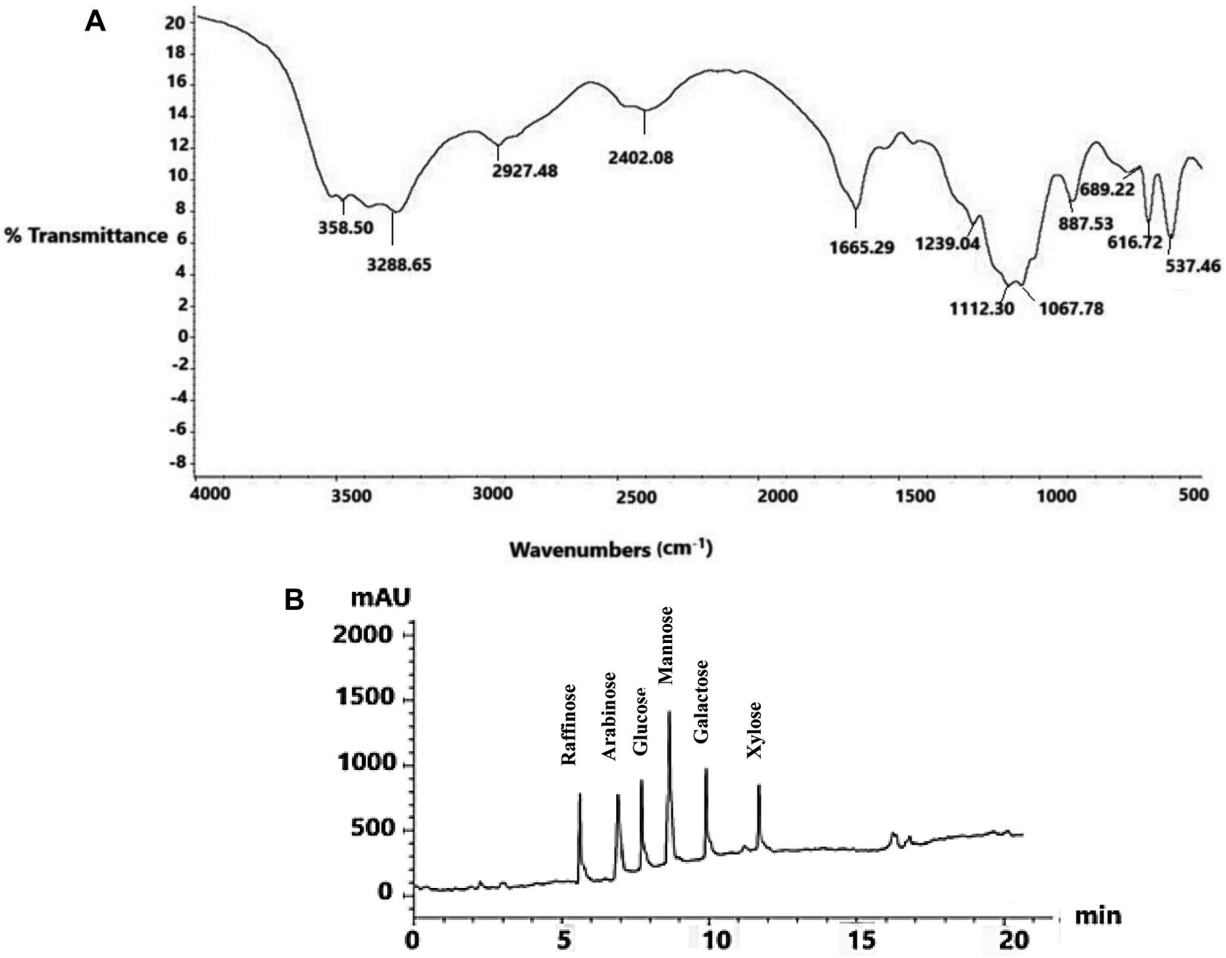
(**A**) FT-IR spectrum of the purified exopolysaccharide from P. aeruginosa AG01. (**B**) HPLC analysis of the purified exopolysaccharide from *P. aeruginosa* AG01

FT-IR spectroscopy was utilized to investigate the functional groups of the partially purified EPS from *P. aeruginosa* AG01. Various bands with differing intensities were identified (Fig. 3A; Table 2). The peaks at 3480.50 cm^− 1^ and 3288.65 cm^− 1^ correspond to the O-H stretching of hydroxyl groups (-OH). The absorption band at 2927.48 cm^− 1^ is attributed to the C-H stretching of methylene groups, while the band at 2402.08 cm^− 1^ may be associated with C-H stretching of methyl or additional methylene groups.

Moreover, the peaks at 1655.29 cm^− 1^ suggest the presence of an N-acetyl group or protonated carboxylic acid (C = O). The band at 1239.04 cm^− 1^ is linked to the carboxyl group (C = O), and the band at 1112.30 cm^− 1^ is associated with O-acetyl ester-linked uronic acid (O-O-acetyl ester). The absorption bands at 1067.78 cm^− 1^ could be related to the anomeric region with pyranose ring structures (C-O-C).

Additionally, the α-D-glucan is represented by the band at 887.53 cm^− 1^. The C-O stretching of the carboxylic acid (COOH) group appears as a thiol group with absorption bands at 689.22 cm^− 1^ and 616.72 cm^− 1^. In addition, the existence of sulfate (O-S-O) is indicated by a strong distinctive absorption at 537.46 cm^− 1^.

**Table 2:** Functional groups in EPS produced by *P. aeruginosa*

Wave number (cm^_1^)	Functional groups	Name of group
3480.50	O-H stretch	Hydroxyl
3288.65	O-H stretch	Hydroxyl
2927.48	C- H stretch	Methylene
2402.08	C- H stretch	Methyl or methylene
1655.29	C = O	N-acetyl group or protonated carboxylic acid
1239.04	C = O	Carboxyl
1112.30	O- acetyl ester	O-acetyl ester linked uronic acid
1067.78	C-O-C	Anomeric region with pyranose ring
887.53	α-D glucan	α-D glucan
689.22	C-O stretching of COOH	Carboxylate
616.72	C-O stretching of COOH	Carboxylate
537.46	O–S–O	Sulfate

### HPLC analysis of EPS

Six peaks were detected by HPLC chromatography of EPS (Fig. 3B). The EPS was found to be monosaccharides consisting of mannose (14.02 mg/g), galactose (9.85 mg/g), glucose (8.56 mg/g), raffinose (8.11 mg/g), arabinose (7.98 mg/g), and xylose (4.68 mg/g).

### Antioxidant activity of EPS

Antioxidants can influence DPPH radical scavenging and ABTS scavenging, which is associated with their capacity to donate hydrogen. The data in Table 3 demonstrate that, in comparison to the controls, ascorbic acid, and butylated hydroxytoluene, increasing concentrations of partially purified EPS (100, 200, 300, and 400 µg/ml) considerably increased the scavenging of DPPH radicals and ABTS activity. The most notable increases in DPPH radical scavenging and ABTS activity were observed at 400 µg/ml EPS, with percentages reaching approximately 99.5% and 70.9%, respectively. The IC50 values for DPPH radical and ABTS scavenging with partially purified EPS were found to be 218.30 µg/ml and 293.77 µg/ml, respectively.

**Table 3:** Antioxidant activities (DPPH and ABTS) of purified EPS produced by *P. aeruginosa* as well as ascorbic acid and butylated hydroxytoluene at different concentrations. The values are the means of three replicates

Treatment	Concentration (µg/ml)	% Inhibition of DPPH	IC50 (μg/ml)	% Inhibition of ABTS	IC50 (μg/ml)
Purified EPS	100	18.7	218.30	12.2	293.77
	200	38.5		32.4	
	300	79.2		50.5	
	400	99.5		70.9	
Ascorbic acid	5	23.5	19.57	32.0	22.92
	10	39.9		34.0	
	20	51.1		48.2	
	40	71.1		68.0	
Butylated hydroxytoluene (BHT)	5	23.1	18.35	37.3	9.58
	10	36.6		52.2	
	20	61.3		67.1	
	40	81.6		90.2	

### Antimicrobial activity of EPS

**Fig. 4:**
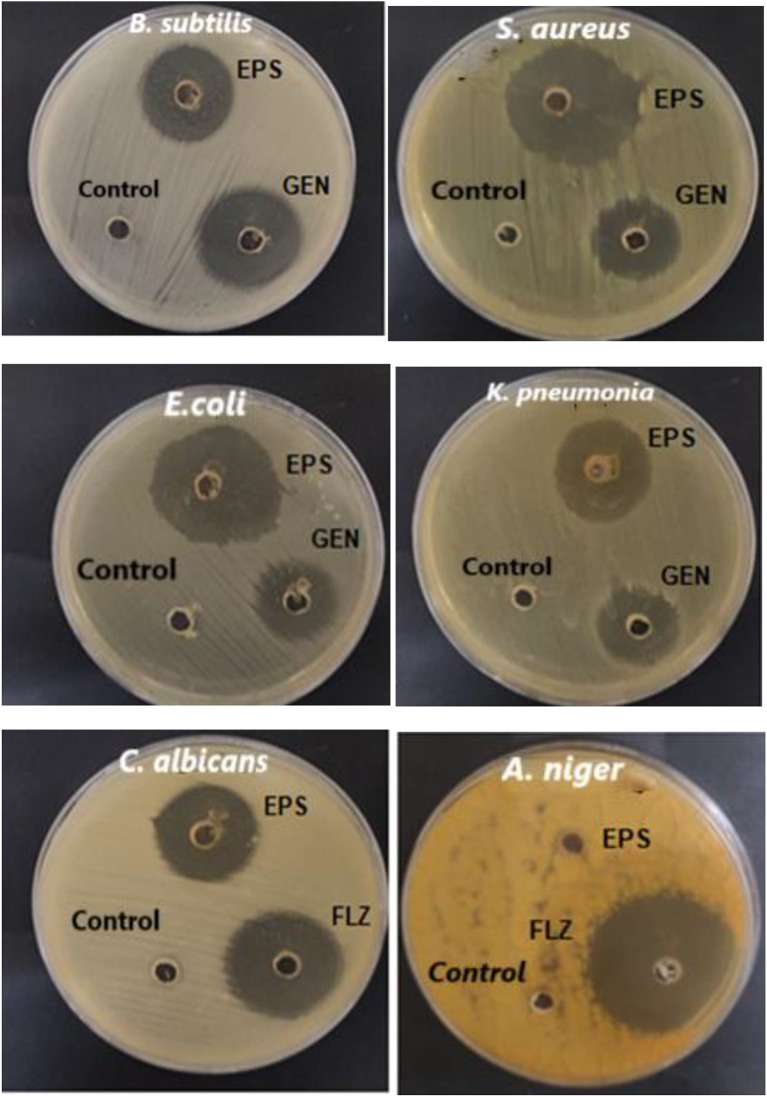
Antimicrobial efficiency of EPS produced from *P. aeruginosa* AG01 against gram‑positive, gram‑negative, and fungi

EPS produced by *P. aeruginosa* AG01 exhibited antimicrobial action against a range of gram-positive and gram-negative bacteria as well as fungi, according to the well diffusion experiment results, which are displayed in Table 4; Fig. 4. In comparison to common medicines like fluconazole and gentamicin, the EPS samples showed differing levels of antibacterial and fungal activity against the tested microorganisms.

The inhibitory zones for *B. subtilis*, *S. aureus*, *E. coli*, *K. pneumoniae*, and *C. albicans* measured approximately 24 mm, 33 mm, 35 mm, 26 mm, and 25 mm in diameter, respectively, when compared to the antibiotics. Notably, the produced EPS exhibited the most significant antibacterial activity (*P* < 0.05) against *E. coli*, followed by *S. aureus* among the tested microorganisms, in comparison to the antibiotic gentamicin. In contrast, the EPS showed no activity against *Aspergillus niger* (ATCC 16888).

**Table 4:** Antimicrobial efficiency of purified EPS produced by *P. aeruginosa* against Gram-positive, Gram-negative, and fungi

Pathogenic microorganism	Inhibition zones of EPS (mm)	Inhibition zones of antibiotic (mm)
Gram +ve bacteria		
*Bacillus subtilis* (ATCC 6633)	24±0.1^e^	27±0.1
*Staphylococcus aureus (ATCC 6538)*	33±0.2^b^	23±0.2
**Gram -ve bacteria**		
*Escherichia coli (ATCC 8739)*	35±0.1^a^	21±0.1
*K. pneumonia (ATCC13883)*	26±0.1^c^	28±0.2
**Fungi**		
*Candida albicans (ATCC 10221)*	25±0.2^d^	28±0.2
*Aspergillus niger* (ATCC 16888)	*NA	3±0.1

The values are the means of three replicates with standard deviation (± SD). Mean values in each column followed by a different lower-case letter are significantly different according to Duncan’s multiple range tests at *p*≤0.05

* NA: No activity. * Control for Bacteria was gentamycin and for fungi was fluconazole at concentration 1.0 mg/ml

### Anti-biofilm assay of EPS

**Fig. 5:**
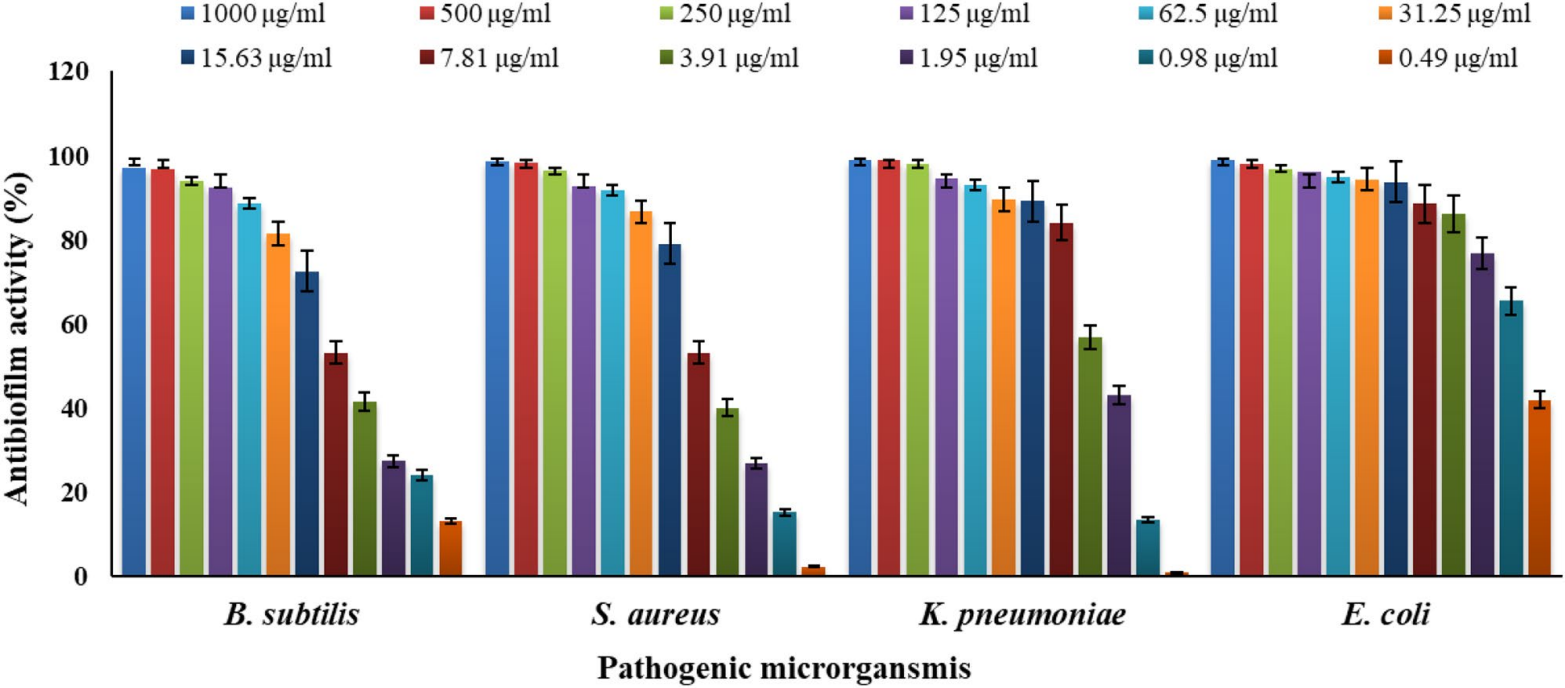
Antibiofilm efficiency of partially purified EPS produces *P. aeruginosa*

In the current study, EPS antibiofilm activity against four biofilm-producer bacteria *S. aureus*, *B. subtilis*, *E. coli*, and *K. pneumonia* were determined. The results in Table 5; Fig. 5 illustrated the maximum suppression of EPS on biofilm formation without sacrificing bacterial viability, with inhibition reaching 98.93%, 98.86%, 98.63%, and 97.19% at 1000 µg/mL and 42.06%, 1.14%, 2.36% and 13.17% at 0.49 µg/mL for *E. coli*,* K. pneumonia*,* S. aureus*, and *B. subtilis*, respectively.

**Table 5:** Antibiofilm efficiency of purified EPSs produce *P. aeruginosa*

Concentrations (µg/ ml)	Mean of O.D (Antibiofilm formation)	Anti-biofilm activity of Bacillus subtilis %	Mean of O. D	Anti-biofilm activity of Staphylococcus aureus %	Mean of O. D	Anti-biofilm activity of Klebsiella pneumoniae %	Mean of O.D	Anti-biofilm activity of Escherichia coli %
Blank (Media only)	0.055±0.004		0.043±0.003		0.038±0.005		0.033±0.004	
Media and Organism (Positive Cont.) Biofilm formation	0.708±0.013	-	0.749±0.006	-	0.829±0.015	0	0.783±0.008	0.00
1000.00	0.073±0.014	97.19	0.053±0.009	98.63	0.047±0.002	98.86	0.041±0.003	98.93
500.00	0.076±0.004	96.73	0.055±0.002	98.25	0.047±0.003	98.90	0.049±0.002	97.91
250.00	0.095±0.003	93.93	0.069±0.003	96.32	0.053±0.002	98.10	0.056±0.004	96.89
125.00	0.104±0.005	92.50	0.095±0.004	92.68	0.081±0.003	94.52	0.062±0.004	96.09
62.50	0.128±0.005	88.77	0.101±0.003	91.74	0.094±0.003	92.88	0.072±0.002	94.75
31.25	0.176±0.007	81.47	0.137±0.005	86.64	0.120±0.003	89.63	0.075±0.011	94.35
15.63	0.235±0.003	72.49	0.191±0.003	79.04	0.123±0.008	89.21	0.080±0.006	93.73
7.81	0.361±0.006	53.19	0.373±0.005	53.26	0.164±0.002	84.03	0.119±0.006	88.57
3.91	0.437±0.006	41.55	0.465±0.006	40.18	0.380±0.013	56.81	0.136±0.008	86.26
1.95	0.529±0.002	27.41	0.559±0.003	26.91	0.488±0.003	43.07	0.207±0.012	76.79
0.98	0.551±0.010	24.04	0.641±0.007	15.30	0.722±0.009	13.53	0.291±0.013	65.58
0.49	0.622±0.009	13.17	0.732±0.008	2.36	0.820±0.008	1.14	0.467±0.013	42.06

The values are the means of three replicates with standard deviation (± SD)

### Anticancer activity of EPS

To evaluate the safety of EPS for humans, the cytotoxicity assay was performed in vitro using normal cell lines (Vero cells). The median value (IC50) for EPS was determined to be between 1000 and 31.25 µg/ml. The results in Table 6; Fig. 6A showed that the IC50 of the EPS was 590.71 ± 5.06 µg/ml and the viability of Vero cell ranged between 95.74% and 99.86% between 250 and 31.25 µg/ml with inhibition rate between 4.25% and 0.14%. So, EPS is regarded as non-cytotoxic.

The data in Table 6; Fig. 6B showed the antiproliferative activity effects of different concentrations of EPS from *P. aeruginosa* AG01 (31.25, 62.5, 125, 250, 500, and 1000 µg/ml) against prostate cancer (PC3). In this investigation, EPS exhibited varying degrees of anticancer efficacy against the studied cancer cell lines, and increasing EPS concentrations caused inhibition in the cell viability. The antiproliferative activity of EPS was present at doses of 250 µg/ml, which significantly inhibits the viability of PC3 cell lines with non-cytotoxic effect on normal cells by about 96.35% with IC50 values of 156.41 ± 1.12 µg/ml (Table 6; Fig. 6B). In the same line, the percentage of inhibition of viable MCF7 cell lines was approximately 85.40% at 250 µg/ml, with an IC50 value of 114.6 ± 1.46 µg/ml, as shown in Table 6; Fig. 6C.

**Fig. 6A:**
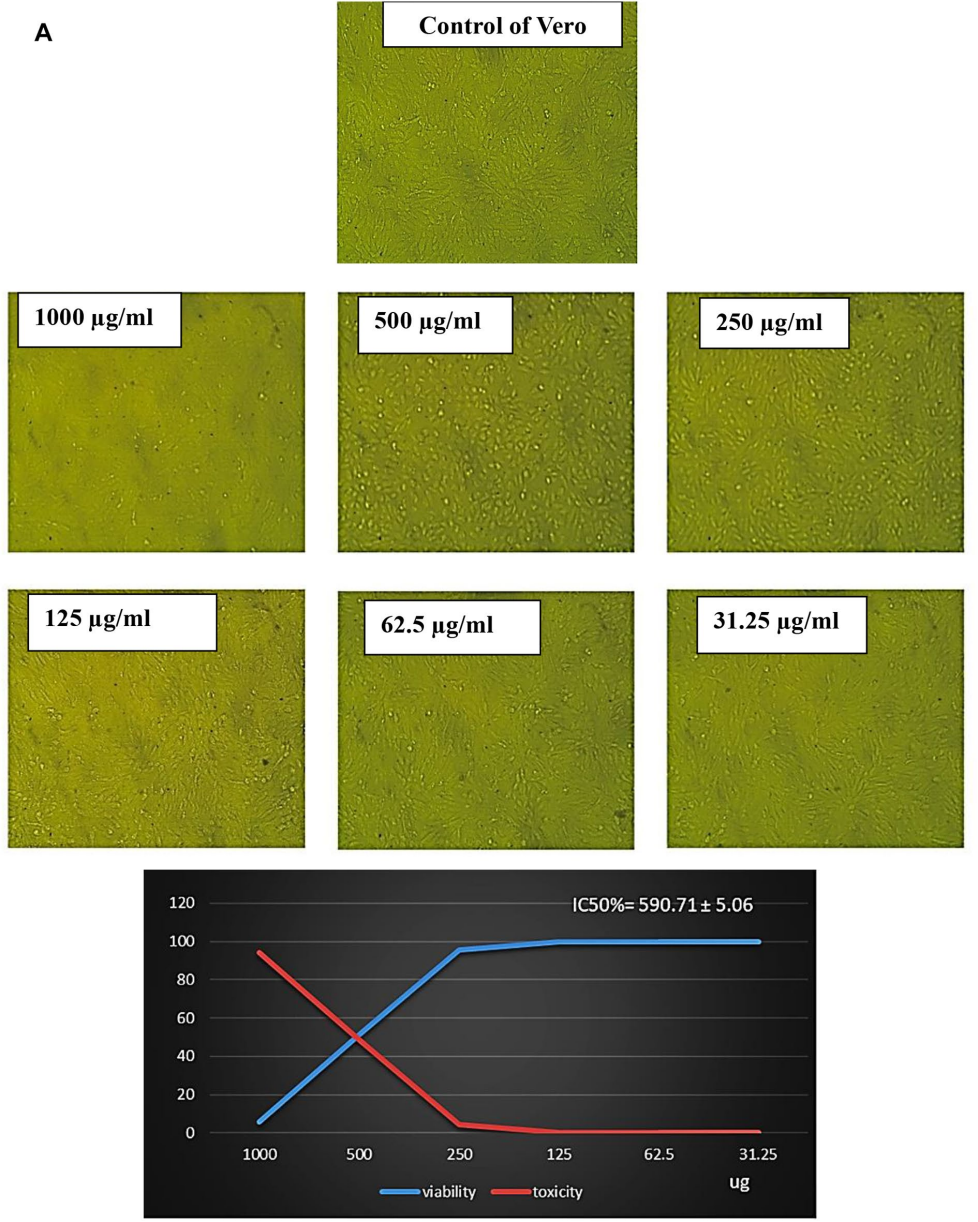
Effect of different concentrations of EPS produced from *P. aeruginosa* AG01 on normal Vero cell line

**Fig. 6B:**
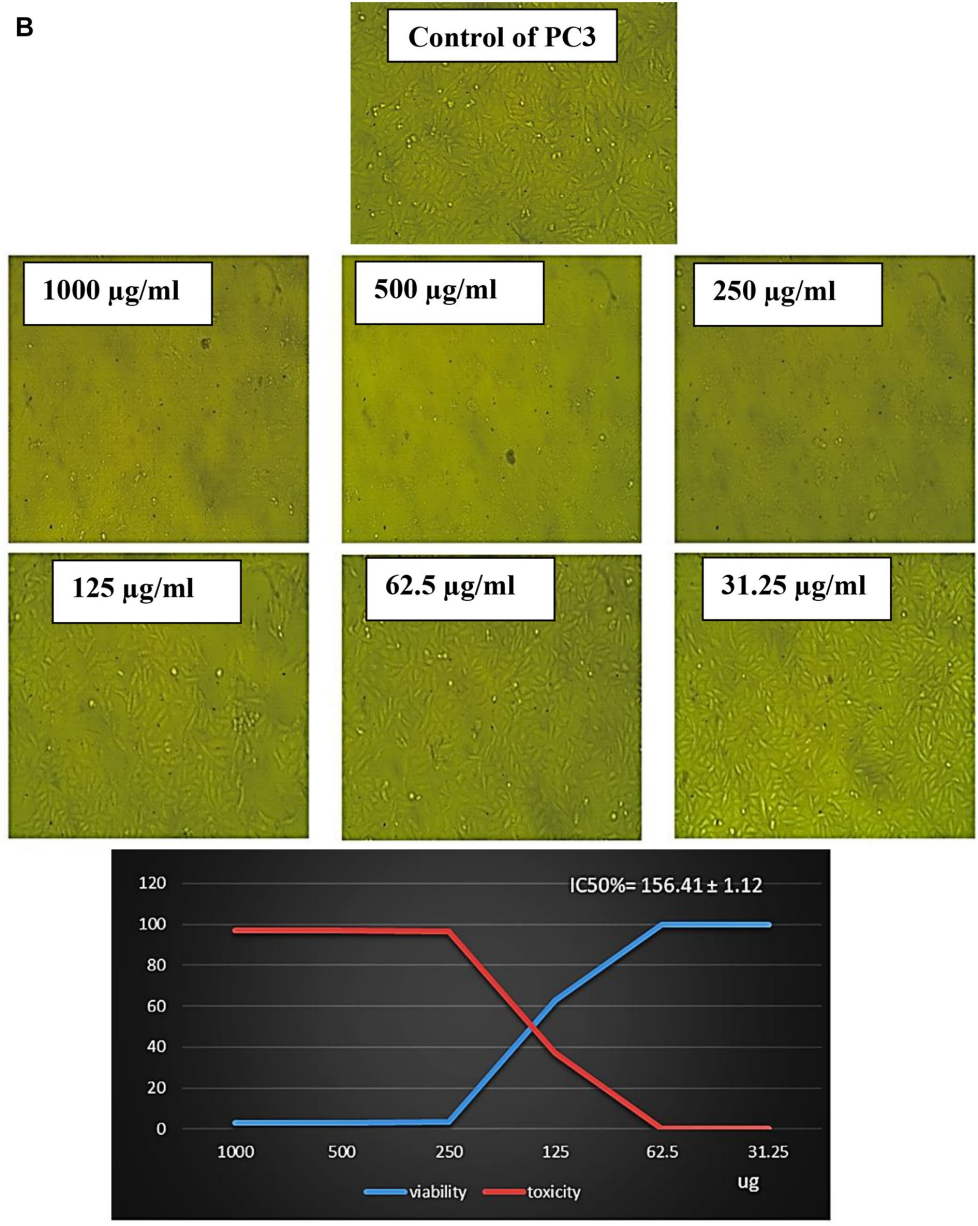
Effect of different concentrations of EPS produced from *P. aeruginosa* AG01 on PC3 cell line

**Fig. 6C:**
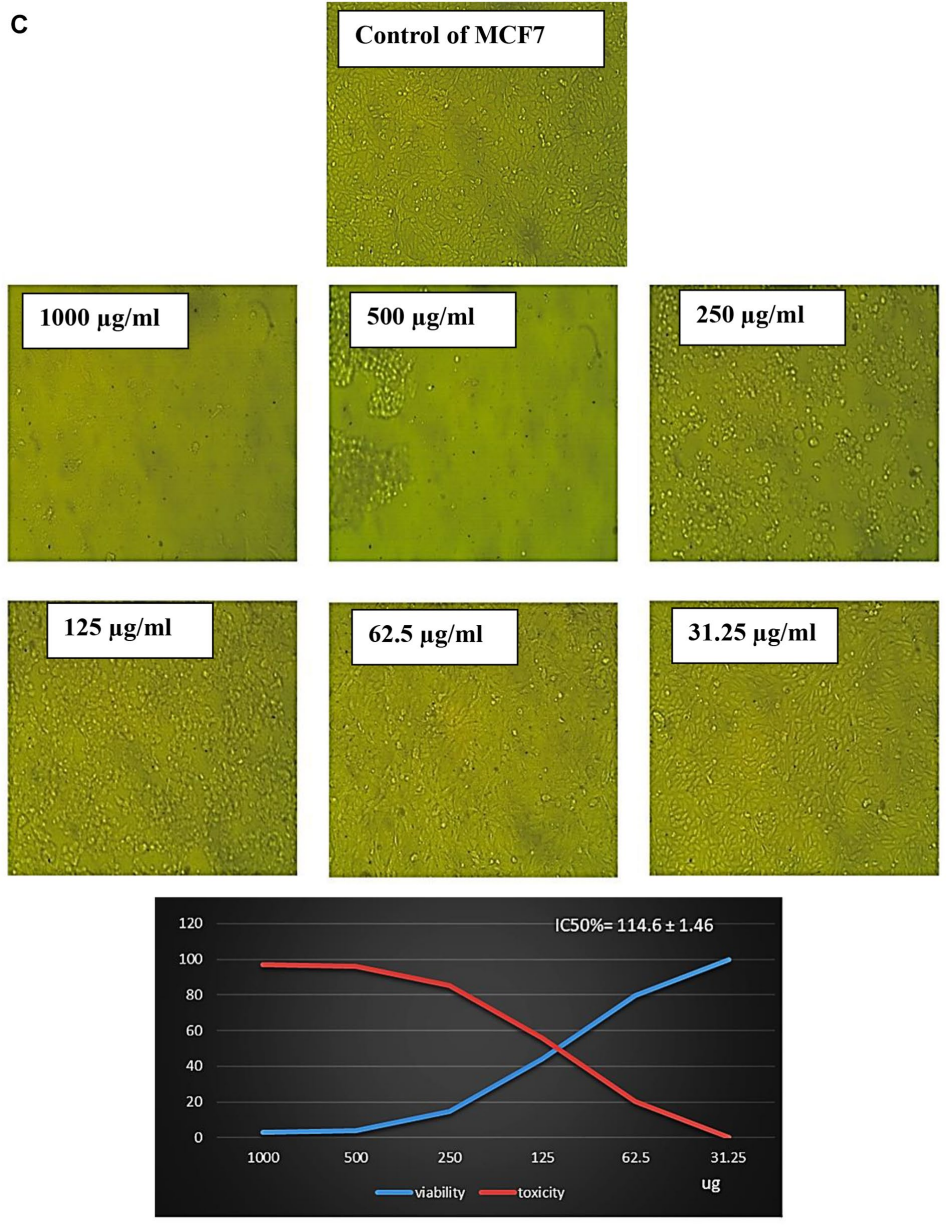
Effect of different concentrations of EPS produced from *P. aeruginosa* AG01 on MCF7 cell line

**Table 6:** Effect of purified EPS produced by *P. aeruginosa* as an anticancer against different cell lines (Vero, PC3, and MCF7)

Cell line	Conc. of purified EPS µg/ml	Mean O. D	ST. E	Viability %	Toxicity %	IC50 µg/ml
	Control	0.696	0.003	100	0	
Vero	1000	0.039	0.001	5.65	94.35	590.71 ± 5.06
	500	0.357	0.005	51.29	48.71	
	250	0.666	0.007	95.74	4.26	
	125	0.695	0.002	99.81	0.19	
	62.5	0.695	0.001	99.81	0.19	
	31.25	0.695	0.002	99.86	0.14	
PC3	Control	0.667	0.0029	100	0	156.41 ± 1.12
	1000	0.019	0.0006	2.85	97.15	
	500	0.019	0.0003	2.90	97.10	
	250	0.024	0.0019	3.65	96.35	
	125	0.415	0.0046	62.77	37.23	
	62.5	0.665	0.0015	99.70	0.300	
	31.25	0.666	0.0021	99.85	0.150	
MCF7	Control	0.751	0.003	100	0	114.6 ± 1.46
	1000	0.022	0.001	2.97	97.03	
	500	0.028	0.001	3.77	96.23	
	250	0.110	0.007	14.60	85.40	
	125	0.332	0.004	44.25	55.75	
	62.5	0.599	0.004	79.72	20.28	
	31.25	0.751	0.001	100	0	

The values are the means of three replicates with standard deviation (±SE)

### Cell cycle analysis by flow cytometry of EPS

**Fig. 7:**
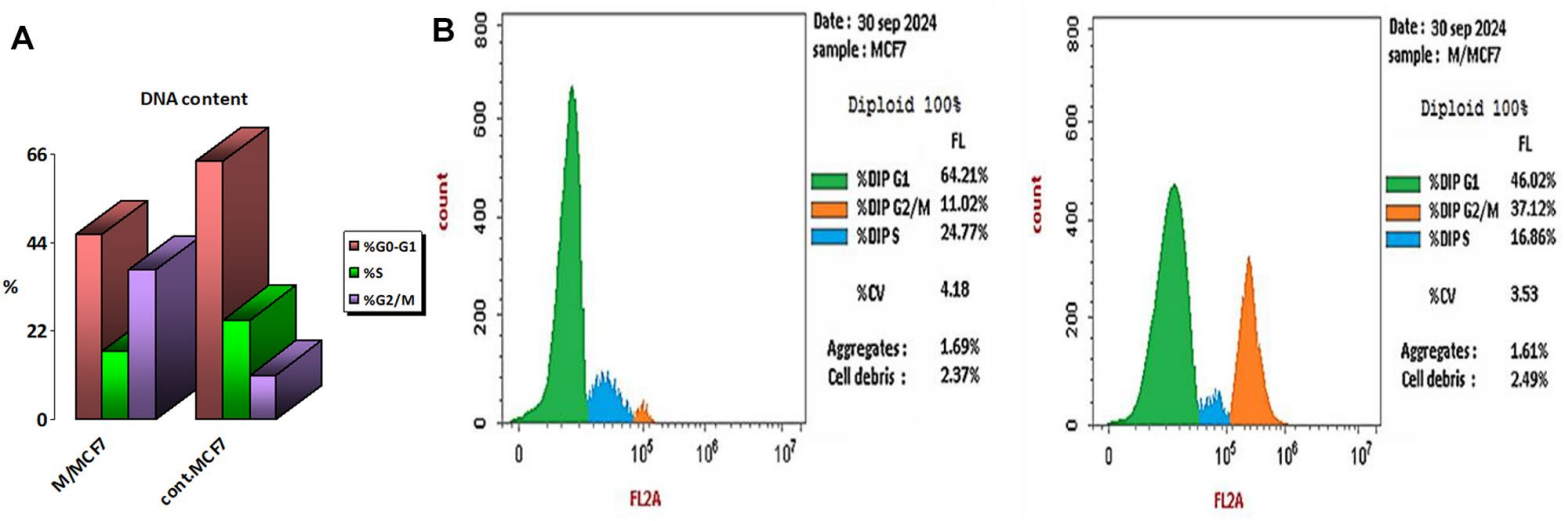
Cell cycle proportions of EPS-treated MCF7 cells: (**A**) The cell cycle of MCF7 cells without or with EPS (250 µg/ml) treatment for 48 h was detected by flow cytometry. (**B**) Quantitative statistics of cells in different periods. Data are expressed as the average of three independent replicates

The cell cycle profiles are based on the distribution of cells according to the structure and cell cycle phase. The changes in DNA content during the cell cycle progression following EPS treatment were examined using flow cytometry. The findings in Fig. 7A-B demonstrated that, in comparison to the untreated control MCF7 cells, MCF7 cells treated with EPS (250 µg/ml) for 48 h had a lower percentage of cells in G0/G1 and S phase proportions. The proportion of treated cells dropped from 24.77 to 16.86% in the S phase and from 64.21 to 46.02% in the G0/G1 phase. However, compared to control cells, which had a G2/M phase percentage of 11.02%, the treated cells’ percentage rose dramatically to approximately 37.12%.

### Apoptosis and necrosis of EPS

**Fig. 8:**
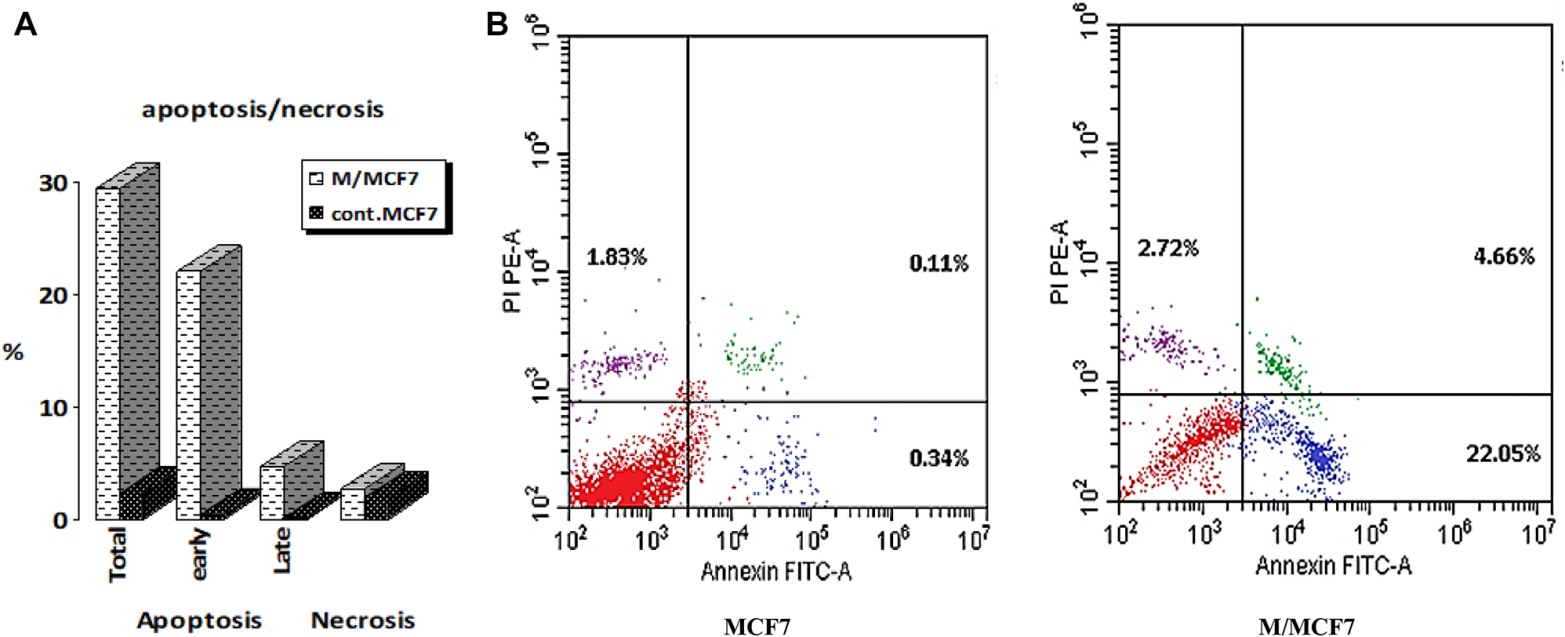
Effect of EPS on the expression level of apoptosis and necrosis (**A**) and flow cytometry analysis with Annexin FITC-A (**B**)

Apoptosis and necrosis analyses were conducted to evaluate the effect of EPS on apoptosis induction in MCF7 cells using the Annexin V-FITC PI staining method. As shown in Figs. 8A-B, treatment with EPS resulted in 22.05% of cells exhibiting early apoptosis and 4.66% exhibiting late apoptosis. The cytotoxic effect of EPS from *P. aeruginosa* AG01 on MCF7 cells was associated with the induction of apoptosis. In contrast, control cells displayed only 0.34% for early apoptosis and 0.11% for late apoptosis. Consequently, the overall percentage of apoptotic cells (including both early and late phases) increased from 2.28% in the control group to 29.43% in the EPS-treated group, representing a 12.9-fold increase. Additionally, only 2.72% of necrotic cells were observed in the EPS-treated MCF7 cells. According to the findings, MCF7 cells treated with EPS exhibited a dose-dependent increase in apoptosis frequency in contrast to MCF7 cells not treated with EPS. Cell necrosis after treatment is thought to be related to DNA fragmentation, given the comparatively high rate of late apoptosis observed. Thus, rather than necrosis, apoptosis is probably the main cause of cell death.

### Antiviral activity of EPS

The purpose of this study was to assess how well EPS inhibited the growth of the hepatitis A virus (HAV) and herpes simplex virus (HSV-1), which can both cause liver and oral infections. Initially, the cytotoxic effects of EPS on a normal Vero cell line were assessed to determine the maximum non-toxic concentration (MNTC), which was found to be 250 µg/ml (Table 7).

The findings indicated that EPS exhibited significant antiviral activity against both HSV-1 and HAV. It was found that EPS was more effective against HAV than HSV1. Specifically, the percentage of viral activity was recorded at approximately 52.77% for HSV-1 and 11.82% for HAV at 250 µg/ml of EPS. Additionally, the overall antiviral activity percentage of EPS was around 47.23% for HSV-1 and 88.18% for HAV, with IC50 values of 263.36 ± 11.55 µg/ml for HSV-1 and 144.61 ± 3.13 µg/ml for HAV (Table 7).

**Table 7:** Effect of purified EPS produced by *P. aeruginosa* as antiviral against different cell lines (HSV1 and HAV)

Cell line	Conc. of purified EPS µg/ml	Mean O. D	ST. E	Viability %	Toxicity %	Viral activity %	Antiviral effect %	IC50%
Vero	Control	0.696	0.002	100	0			590.71 ± 5.06
	1000	0.039333	0.001	5.65	94.34			
	500	0.357	0.005	51.29	48.70			
	250	0.666333	0.007	95.74	4.26			
	125	0.694667	0.0018	99.80	0.19			
	62.5	0.694667	0.0012	99.82	0.19			
	31.25	0.695	0.002	99.84	0.14			
HSV1	Control	0.325	0.004	45.37	54.63	100	0	263.36 ± 11.55
	250	0.510	0.007	71.18	28.82	52.77	47.23	
	125	0.391	0.006	54.58	45.42	83.15	16.85	
	62.5	0.342	0.003	47.65	52.35	95.83	4.17	
	31.25	0.333	0.002	46.44	53.56	98.04	1.96	
HAV	Control	0.350	0.003	48.86	51.14	100	0	144.61 ± 3.13
	250	0.674	0.004	93.96	6.04	11.82	88.18	
	125	0.538	0.005	75.08	24.92	48.73	51.27	
	62.5	0.395	0.002	55.04	44.96	87.91	12.09	
	31.25	0.367	0.001	51.19	48.81	95.45	4.545	

The values are the means of three replicates with standard deviation (±SE)

## Discussion

Microbial exopolysaccharides have been widely studied for their potential biomedical and pharmaceutical applications [47–48]. Following ethanol precipitation, the EPS in this study yielded 0.89 g/L of EPS in nutrient broth media. These findings are consistent with those of Kılıç and Dönmez [49], who found that *P. aeruginosa* produced the largest amount of EPS (0.863 g/L) during 96 h of incubation at 20 °C on media containing 50 mg/L Cr (VI).

The production of EPS depends on carbon sources, specific microbial strains, the growth phase of microbes, culture conditions such as pH and temperature, and the duration of incubation [50]. The results of the current investigation showed that the optimal conditions for culturing *P. aeruginosa* AG01 for EPS production were determined to be a temperature of 32 °C, an initial pH of 6, an incubation time of 96 h, glucose as carbon source, and yeast extract and peptone as nitrogen source. The production of EPS in this study is affected by the growth phase of the organism. The results indicated that the highest EPS yield, measured at 38.5 mg/50 ml, was observed after 96 h at a pH of 7. This yield remained stable, showing no significant difference up to 120 h. Additionally, both the amount of biomass and EPS increased during the logarithmic phase, with the maximum amounts recorded during the stationary phase. These results are similar to Chug et al. [32], who found that *P. aeruginosa* MTCC 1688 produces 26 mg/50 ml of EPS after 96 h of incubation at 32 ℃ (pH 6) in nutrient broth.

Several culture parameters, including temperature and pH, affect the synthesis of EPS in static cultures in addition to the incubation time. An important consideration is the growth medium’s initial pH, which affects EPS production, nutrient uptake, and cell growth [48]. In this study, it was determined that the optimal EPS production by *P. aeruginosa* AG01 occurred at an initial pH of 6 and a temperature of 32 °C. Any further increase in temperature or pH resulted in decreased cell growth and EPS yield. These results align with those of Chug et al. [32], who discovered that 32 °C is the best temperature for reaching the highest EPS yield and that pH 6 is optimal for optimizing EPS production (23.3 mg/50 mL).

The media with galactose and glucose showed the highest EPS yield in the current investigation. In contrast, the lowest EPS production was found in nutrient broth media with lactose. Similarly, Lee et al. [52] reported that a medium containing galactose produced the highest extracellular polysaccharide by *Pseudomonas* sp. GP32. Similarly, Arayes et al. [53] found that glucose gave the highest yield of EPS from *Alkalibacillus sp.* Glucose and galactose are also recognized as effective substrates for the production of extracellular polysaccharides. Nitrogen sources play a crucial role in the biosynthesis of EPS and cell growth [54]. The results indicate that the highest yield of EPS was achieved using yeast extract and peptone, followed by beef extract. In contrast, the lowest EPS production occurred in the nutrient broth medium that contained ammonium sulfate. These results are incompatible with Maalej et al. [33], who reported that yeast extract was identified as the most effective nitrogen source for EPS production by *P. stutzeri* AS22. Moreover, Raza et al. [55] also reported that the optimal source of nitrogen for the production of EPS from *P. fluorescens* WR-1 was peptone. Peptone and yeast extract are examples of organic nitrogen sources. These are complex nutrients that include purines, pyrimidines, vitamins, and minerals. These elements might be essential for encouraging cell division and raising EPS yield [54]. Our research has led us to conclude that the best sources of nitrogen are organic ones. This is probably due to the inability of several important amino acids to be produced from inorganic nitrogen molecules, which restricts growth and lowers the generation of EPS [56].

Bacterial exopolysaccharides are defined by the presence of various functional groups embedded in their matrix, including amino groups, amides, carboxylic acids, hydroxyl groups, and phosphates. These functional groups are essential components of EPS, enabling the molecules to undergo modifications that confer new and valuable properties [29, 57]. The FT-IR spectrum of the EPS in this study displayed a broad stretching peak at 3480.50 cm^− 1^ and a second peak at 3288.65 cm^− 1^. These peaks correspond to the stretching vibrations of hydroxyl groups, which are characteristic of a carbohydrate ring [58]. Additionally, two weak bands were observed at 2927.48 cm^− 1^ and 2402.08 cm^− 1^. These bands can be attributed to the C-H stretching of methyl or methylene groups, commonly found in hexoses such as glucose or galactose, as well as in deoxyhexoses like rhamnose or fucose [58–59]. The peak at 1655 cm^− 1^ corresponds to a C-O stretching vibration of the N-acetyl group or a protonated carboxylic acid [59]. The FT-IR spectra of the polymer show a carboxylic group in the region around 1239.04 cm^− 1^, which implies that it might serve as a binding site for divalent cations. Additionally, this group could serve as a functional moiety for creating a new or modified polymer [60]. Additionally, the carboxyl group might serve as a metal ion binding site, which has been suggested as a possible mechanism for antimicrobial activity [61].

The presence of the carboxylic group indicates that the sample contains amino sugars [60]. The absorption band typically corresponds to the stretching vibration of the C = O group [63]. Moreover, the peak observed at 1112.30 cm^− 1^ is likely associated with O-acetyl ester-linked uronic acid [59]. The strong absorption at 1067.78 cm^− 1^, found within the range of 1200 to 1000 cm^− 1^, is attributed to the C-O-C and C-O groups present in polysaccharides. This suggests that the monosaccharides in the EPS have a pyranose ring structure [59]. The band observed at 887.53 cm^− 1^ is characteristic of α-D-glucan. Additionally, the small peak at 537.46 cm^− 1^ indicates the presence of a sulfated group [58]. Uronic acids are among the complexly structured sulfated polysaccharides found in EPS, according to FT-IR band assignments. It is well-recognized that sulfated exopolysaccharide derivatives have advantageous qualities, especially as medicinal agents [59].

In the present study, the EPS was found to be monosaccharides consisting of mannose, galactose, glucose, raffinose, arabinose, and xylose. These results align with the findings of Zaghloul and Ibrahim [64], who reported that GC-MS analyses identified five monosaccharides: rhamnose, galactose, mannose, glucose, and arabinose.

The DPPH radical is utilized to assess the antioxidative activity of antioxidants [37]. The partly purified EPS made from *P. aeruginosa* AG01 in this study exhibits antioxidant activity in comparison to the standards of ascorbic acid and BHT. Because of their important function in scavenging free radicals and preventing oxidative damage in living things, EPS may be investigated as a possible new antioxidant. These outcomes are consistent with those of Wang et al. [65], who found that EPS derived from *Lactobacillus plantarum* KX041 showed higher antioxidant activity against free radicals such as hydroxyl, superoxide, DPPH, and ABTS. Numerous methods, such as the binding of transition metal ion catalysts, free radical scavenging, inhibition of chain initiation, and reductive capacity, have been used to assess the antioxidant activity of EPS [66].

Gram-positive and gram-negative bacteria, as well as fungi are more susceptible to EPS than to antibiotics, according to the results of the antibacterial activity of EPS produced by *P. aeruginosa* AG01. Furthermore, it was discovered that EPS had a stronger antibacterial impact than the common antibiotic Gentamicin against *S. aureus* and *E. coli*. Furthermore, the EPS from *B. altitudinis* showed a broad-spectrum impact against all examined microorganisms, including both bacteria and fungi [36]. Likewise, EPS produced from *Alkalibacillus* sp. w3 has shown antimicrobial efficacy against both Gram-positive and Gram-negative bacteria, as well as the yeast *C. albicans* [50]. Numerous functional groups, including carbonyl, phosphate, and hydroxyl groups, have been found in EPS and are thought to be essential for exhibiting antibacterial, antioxidant, and anticancer properties [67]. It has been proposed that EPS disrupts cell division, preventing cell proliferation and causing damage to the cytoplasmic membrane, cell walls, and DNA [68]. One possible explanation for EPS’s inhibitory action against Gram-positive bacteria is its interactions with murein, a crucial part of their cell walls. Furthermore, EPS might interfere with the enzymes that produce peptidoglycans, which would hinder the correct development of the cell wall and ultimately result in cell death [69].

Sivasankar et al. [70] proposed that because Gram-positive bacteria have a stronger positive charge on their cell walls, negatively charged EPS, which is linked to the sulfate group, can interact with them more effectively. Gram-negative bacteria’s outer membranes may have channels or receptors blocked by this contact. Furthermore, research on EPS against *Candida* indicated that the EPS layer could significantly decrease hyphal formation, prevent fungal adhesion, and reduce the availability of nutrients [71]. Rajoka et al. [66] suggested three mechanisms of antibacterial activity: (1) ionic surface interaction that disrupts the cell wall, (2) metal chelation, which creates an external barrier and suppresses vital nutrients for microbial growth, and (3) inhibition of mRNA and protein synthesis by the penetration of bioactive compounds into bacterial DNA.

Biofilms are complex bacterial communities covered in an extracellular matrix consisting of proteins, nucleic acids, and polysaccharides. Because these bacterial biofilms give the bacteria a barrier to protect them from environmental stressors and the host’s immunological response, they are the source of persistent and recurring infections. The bacteria’s resistance to antibiotics and other antimicrobial agents is also improved by biofilms [69]. The present investigation demonstrated that the EPS of *P. aeruginosa* AG01 had antibiofilm action against four bacteria that generate biofilms: *S. aureus*,* B. subtilis*,* E. coli*, and *K. pneumonia*. These results are similar to their findings, suggesting that EPS may be used to treat infections linked to biofilms. Through the inhibition of cell-to-cell surface contacts and the weakening of their cell surface modifications, EPS decreased the initial attachment and auto-aggregation of harmful bacterial cells. Werning et al. [73] and Srinivash et al. [74] found that EPS can effectively reduce or inhibit biofilm formation by a variety of Gram-positive and Gram-negative pathogens. EPS can also function as signaling molecules that suppress the expression of genes linked to the production of biofilms, which can help prevent or eradicate illnesses caused by biofilms [73]. According to Srinivash et al. [74], the treated biofilm’s visualization showed that the antimicrobials had successfully penetrated the biofilm matrix and caused cell death. Quorum sensing is a cellular mechanism that depends on bacterial population density, whereby bacteria produce chemical signals to communicate within their environment. Exopolysaccharides (EPS) may serve as potential anti-biofilm agents. Research has shown that EPS exhibits biofilm inhibition activity against a variety of both Gram-positive and Gram-negative bacteria [75]. The interference with quorum sensing (QS) results in the downregulation of genes regulated by QS, including those responsible for synthesizing the biofilm matrix, motility, and adhesion. As a result, extracellular polymeric substances (EPS) not only inhibit initial attachment but also hinder the maturation and maintenance of biofilms. These findings suggest that EPS may function as quorum-quenching agents, providing a non-bactericidal approach to controlling biofilm-associated infections by disrupting signals rather than directly killing microbes [75]. Transcriptome analysis suggests that EPS significantly influences the expression of genes related to curli production and chemotaxis. This action disrupts initial attachment, leading to auto-aggregation of bacterial cells by weakening cell surface modifications or by reducing cell-to-cell interactions [75]. Additionally, another mechanism proposed by Barzegari et al. [76] involves competitive inhibition, where biofilm formation by lactic acid bacteria (LAB) can hinder the biofilm development of pathogenic bacteria by competing for nutrients and surfaces to adhere to.

Recently, EPS has shown great potential as an antitumor drug, as new treatment strategies have revealed limitations such as severe side effects and the occurrence of multidrug resistance [77]. In this study, the safety of EPS for humans was evaluated using normal Vero cells. The findings showed that, following a 48-hour exposure, EPS significantly and dose-dependently cytotoxically affected cancer cells. Despite being widely thought of as non-cytotoxic, at 250 µg/ml, the most significant inhibitory effect was detected in PC3 cells, followed by MCF7 cells. Similarly, EPS from *L. hircilactis* showed no effect on the control cells but substantial cytotoxic effects on HT-29 colon cancer cells [74]. Additionally, pure EPS demonstrated anticancer efficacy against human colon cancer (HCT-116) and hepatocellular carcinoma (HepG2) cell lines [53]. Several studies have demonstrated the anticancer properties of polysaccharides, with the mechanisms of inhibition being summed up as follows: immune system activation and augmentation, apoptosis induction, angiogenesis limitation, and stopping the cell cycle [78–79].

The effect of EPS on IC50 of cell lines must be due to several biochemical pathways converging during apoptosis, resulting in the activation of a family of cysteine-dependent aspartate-directed proteases (caspases). Although caspase-dependent or caspase-independent mechanisms can regulate apoptosis, the latter is more prevalent because the majority of cells initiate apoptosis via caspase activation. Board proteins, such as *BCL2*, regulate both cell damage and proliferation. *BCL2* family proteins are required for mitochondria-mediated apoptosis as they maintain the mitochondrial membrane’s integrity [80]. The mechanism by which polysaccharides induce apoptosis in cancerous cells was investigated using *BCL2* family proteins such as *BCL2*, which inhibits apoptosis, and Bax, which induces apoptosis. Numerous studies have shown that tumor cells treated with LAB EPS expressed significantly less *BCL2*. Mahgoub et al. [81] found that EPS decreased the level of *BCL2* while increasing the level of Bax in MCF-7 cells. This suggests that EPS may activate mitochondria-mediated apoptosis by enhancing the permeability of the mitochondrial membrane. Additionally, EPS triggers the release of cytochrome c from the mitochondria into the cytosol. The difference in antitumor activity of bacterial exopolysaccharides (EPS) between PC3 and MCF7 cells is likely due to multiple factors. These factors include hormone receptor status, p53 functionality, membrane transporters, oxidative stress response, and metabolic phenotype. By understanding these mechanisms, we can gain insight into the selective cytotoxicity of EPS, which encourages further exploration of its potential as a targeted cancer therapy [81].

The increased growth rate is a well-known characteristic of cancer cells. Consequently, any agent capable of halting the cell cycle in cancer cells represents an effective anti-cancer treatment. The cell cycle phase is now a target for cancer treatment due to improvements in our knowledge of the processes behind tumor growth and apoptosis induction [82]. In this investigation, we found that the distribution of cells in the G1 and G2/M phases was different from that of the control cells under the conditions of EPS administration. In comparison to the control group, the results showed that EPS administration increased the number of cells in the G2/M phase while decreasing the number of cells in the G1 and S phases. These results imply that EPS inhibits MCF7 cells’ ability to proliferate by blocking their passage from the S phase to the G2/M phase, which results in cell growth arrest at that stage. The findings are consistent with Zhang et al. [83], which demonstrated that EPS administration reduced the proportion of cells in the G0/G1 phase. The proportions of cells in the G2/M and S phases increased. By causing G0/G1 cell cycle arrest in vitro, EPS generated by *Lacticaseibacillus casei* and *L. rhamnosus* was reported by Di et al. [84] to impede the proliferation of HT-29 cells. Generally, the cell cycle and apoptosis govern the control of cell growth [85]. The G0/G1, S, or G2/M phases of the cell cycle are inhibited by several anticancer medications, which cause apoptosis [86].

In this study, apoptosis in MCF7 cells was quantitatively analyzed using flow cytometry. The results demonstrated that 29.43% of the cells underwent apoptosis, indicating a 12.9-fold increase compared to the control group, which showed only 2.28% cell death. Additionally, only 2.72% of the cells treated with EPS were found to be necrotic. According to Zhou et al. [78], the EPS from *Lactobacillus plantarum* NCU116 suppressed the growth of CT26 cells through an apoptotic pathway involving Fas and Fas ligand that was dependent on TLR2 and c-Jun. Additionally, the activation of apoptosis was associated with the cytotoxic action of EPS produced from *Streptococcus thermophilus* CH9 on HepG2 cells [87]. Additionally, it was shown by Emam et al. [40] that exopolysaccharide caused the greatest rate of apoptosis in MCF-7 cells, whilst HepG2 cells showed the lowest rate. In addition, MCF-7 cells showed the most necrosis brought on by EPSs, followed by HepG2 and Caco-2 cells, in that order. Apoptosis induction could be a key cytotoxic mechanism. Accordingly, apoptosis rather than necrosis is probably the main cause of cell death [83].

The anti-cancer properties of extracellular polysaccharides (EPS) may be attributed to several mechanisms: (1) preventing tumor formation, (2) inducing apoptosis in cancer cells, and (3) enhancing immune activity [88]. Apoptosis is crucial for immunomodulation and disease defense, as seen in conditions like colorectal cancer. Typically, caspase-dependent apoptosis is initiated by various external or internal factors [88]. The external pathway is activated through death receptors, which are transmembrane proteins such as Fas and TNF receptors. These receptors bind to their respective ligands, Fas and TNF [88]. When a ligand binds to death receptors, the cytoplasmic domains of these receptors recruit adaptor molecules, which initiate a cascade involving caspases. This process ultimately activates Caspase-8, which in turn activates downstream caspases, such as Caspase-9 and Caspase-3. In the intrinsic pathway of apoptosis, the mitochondrial membrane becomes destabilized, leading to the release of apoptosis-associated proteins like cytochrome c. Cytochrome c, in conjunction with APAF-1, activates Caspase-9, which subsequently activates Caspase-3, resulting in cell apoptosis [88].

The need to create new methods for managing and curing viral infections is urgent. Furthermore, several synthetic and natural substances have been discovered recently for the treatment and prevention of viral diseases [89]. EPS is thought to be a safe, non-toxic, acellular probiotic with particular medicinal benefits [90]. It is well known that bacterial exopolysaccharides have strong antiviral properties. They can release infectious virus particles, decrease virus titers, stop viral DNA replication, and destroy viral particles. Nevertheless, not much research has been done on these compounds’ antiviral potential. The current investigation demonstrated EPS’s antiviral efficacy against HSV-1 and HAV. Furthermore, when the cells were treated with EPS, the virus was completely (100%) adsorbed, which prevented the production and release of infectious HAdV-5 particles. This indicates that compound 26a has potential as an anti-adenoviral drug and has outstanding anti-HAdV-5 action [90].

By preventing viruses from adhering to host cells, polysaccharides can aid in the prevention of viral infections. They accomplish this by interacting with either the viral particles or the host cells. Sulfated polysaccharides are among the polysaccharides that have shown strong antiviral activity. Sulfated polysaccharides have been found to have antiviral properties and to reduce viral adherence against a variety of viruses, such as the herpes simplex virus, influenza virus, hepatitis B virus, and human cytomegalovirus (CMV). It was recently discovered that *Weissella paramesenteroides* MN2C2 sulfated exopolysaccharides exhibited potent anti-Coxsackie virus action [91]. Most antiviral medications function as protease inhibitors, reverse transcriptases, or DNA polymerase inhibitors. Although these antiviral drugs’ exact modes of action are unknown, polysaccharides have been shown to help lower viral infections by preventing viruses from adhering to host cells. This is important since one of the first stages of viral infection is the adsorption of viruses onto host cells [92].

## Conclusion

The maximum EPS produced from *P. aeruginosa* AG01 was seen during the 96-hour incubation period at a pH of 6 and a temperature of 32 ℃. Furthermore, suitable carbon (galactose and glucose) and nitrogen sources (yeast extract and peptone) may promote faster EPS production. FT-IR represents functional groups, including amino groups, amides, carboxylic acids, hydroxyl groups, and phosphates. The partially purified EPS produced from *P. aeruginosa* AG01 has antioxidant activity and antimicrobial activity against various gram-positive bacteria, gram-negative bacteria, and fungi. In addition, EPS showed antibiofilm activity against four biofilm-producing bacteria *S. aureus*, *B. subtilis*, *E. coli*, and *K. pneumonia.* Also, EPS has anticancer activity against PC3 and MCF7 cells and antiviral activity against HSV-1 and HAV. In conclusion, exopolysaccharides are recognized as non-toxic and natural substances. The future of microbial EPS in the biomedical and pharmaceutical industries appears promising, offering the potential for new therapies and treatments that could enhance human health. To achieve industrial-scale production, strategies should optimize both the bioprocess and the fermentation system. Key approaches include scaling up fermentor-based production by selecting robust microbial strains that are suitable for high-density cultivation, enhancing process control (such as pH, oxygen, and temperature), and employing fed-batch or continuous fermentation techniques. Additionally, optimizing substrates involves using cost-effective, renewable feedstocks, such as agricultural waste or lignocellulosic biomass, and tailoring the nutrient composition to maximize yield and productivity. Integrating metabolic engineering and process modeling can further enhance efficiency and scalability.

## Electronic supplementary material

Below is the link to the electronic supplementary material.

Supplementary Material 1

## Data Availability

The data sets generated and analyzed in this study are available from the corresponding author on reasonable request.
