# Supplementary material for: Production and characterization of exopolysaccharides from Pseudomonas aeruginosa AG01 with some medical potential applications
Source: Microb Cell Fact. 2025 May 14;24:107. doi: 10.1186/s12934-025-02730-z (PMC12077034; doi:10.1186/s12934-025-02730-z)
Supplement: Supplementary file 1 — Supplementary Material 1 [file 12934_2025_2730_MOESM1_ESM.docx]

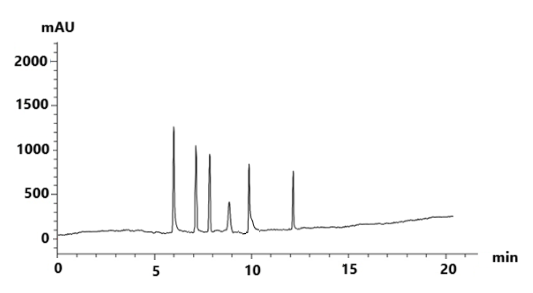


**Rhamnose**

**Galactose**

**Glucose**

**Mannose**

**Arabinose**

**Raffinose**

**Supplemented Figure 1** Standard curve of monosaccharides using HPLC chromatograph.
